# Cerebral and Portal Venous Air Embolism: A Complication of PICC Line Placement

**DOI:** 10.1155/crra/5236025

**Published:** 2025-07-18

**Authors:** Gizem Reyhanoglu, Dominika Moscicka, George Guirguis, Mina S. Mousa

**Affiliations:** ^1^Florida State University Internal Medicine Residency Program, Tallahassee Memorial Hospital, Tallahassee, Florida, USA; ^2^Florida State University College of Medicine, Tallahassee, Florida, USA; ^3^Radiology Associates of Tallahassee, Tallahassee, Florida, USA; ^4^Nova Southeastern University Kiran Patel College of Osteopathic Medicine, Clearwater, Florida, USA

## Abstract

Cerebral air embolism is a rare and potentially fatal medical condition that requires prompt recognition and management. Iatrogenic causes such as laparoscopic procedures, hysteroscopy, or central venous catheter (CVC) manipulation are well-documented etiologies. This article presents a case of an 80-year-old female who developed iatrogenic air emboli from CVC manipulation leading to left middle cerebral artery (MCA) syndrome due to suspected right-to-left shunt from pulmonary arteriovenous malformation (pulmonary AVM) or patent foramen ovale (PFO). Preexisting neurological deficits, elevated lactate levels, and sepsis with evidence of systemic hypoperfusion on admission hindered the early detection and treatment of air emboli. This case highlights the need for heightened awareness of CVC-related iatrogenic air embolism, particularly in patients with predisposing conditions such as pulmonary AVM and PFO. Comprehensive treatment strategies, including hyperbaric oxygen therapy, remain critical for achieving better outcomes.

## 1. Introduction

Cerebral air embolism is an infrequent but serious medical condition that arises from air entry into the vascular system. Iatrogenic causes, including CVC manipulation, laparoscopic procedures, and hysteroscopy, are among the most common triggers [1, 2]. Clinical manifestations can vary widely, ranging from asymptomatic cases or subtle neurological deficits to life-threatening hemodynamic instability, depending on the volume of air and the route it takes within the circulation [3]. Recent evidence by Červeňák et al. highlights that early recognition and aggressive management significantly influence prognosis and functional outcomes in cerebral air embolism, highlighting the critical need for timely diagnosis in high-risk patients [4]. The presence of a right-to-left vascular shunt, such as a PFO or pulmonary AVM, is considered the most serious comorbidity for air emboli, as it significantly increases the risk of developing systemic arterial air embolism by bypassing the pulmonary filtration system [5, 6]. This report presents a case of cerebral air embolism complicated by portal venous gas, emphasizing the interplay of congenital anomalies, predisposing comorbidities, and procedure-related iatrogenic complications in the clinical management of air emboli.

## 2. Case Presentation

**Figure 1:**
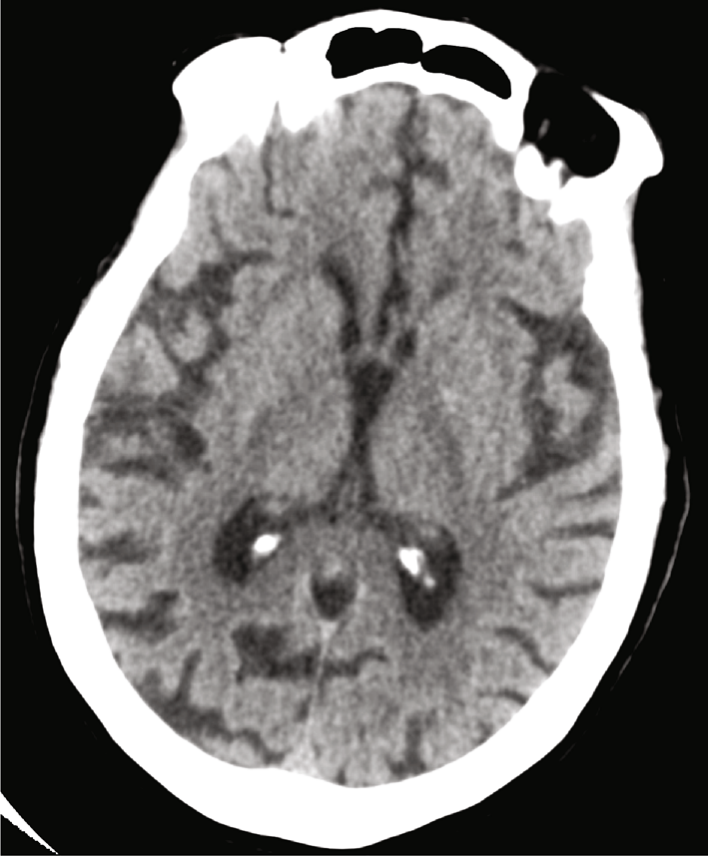
Axial computed tomography image of the head performed without contrast on admission demonstrates normal attenuation of the brain parenchyma, gray-white matter differentiation, without evidence of intracranial hemorrhage, vasogenic edema, mass effect, or midline shift.

**Figure 2:**
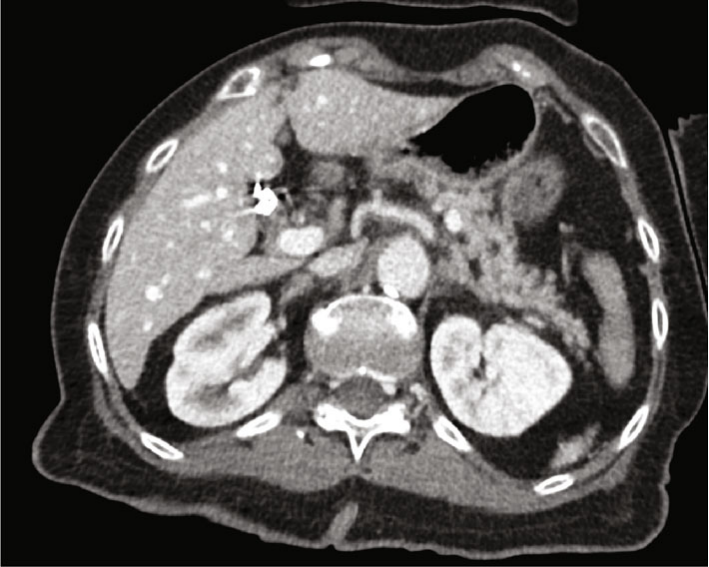
Axial computed tomography image of the upper abdomen obtained on admission demonstrates normal attenuation and enhancement of the liver parenchyma, hepatic veins, and portal vein branches with no evidence of portal venous gas identified. The remainder of the CT abdomen and pelvis exam was negative for source of infection to explain the patient's sepsis clinical presentation.

An 80-year-old female with no significant past medical history presented to the emergency department with fever, leukocytosis, and hypotension (91/78 mmHg), concerning for septic shock. The patient required norepinephrine for blood pressure support and was started on empiric antibiotic therapy per the sepsis protocol, including vancomycin and piperacillin-tazobactam. Initial imaging in the emergency department included a noncontrast CT head that was negative for any acute intracranial processes (Figure 1). Abdominal CT of the upper abdomen was also negative for any acute pathologies (Figure 2). A chest radiograph showed right lower lobe patchy opacity suspicious for aspiration pneumonia, which was the presumed source of sepsis. On admission, the patient had a fever of 101.8°F, lactic acid of 7 mmol/L (normal < 2.1), high sensitivity troponin 18 ng/L (range 0–49.9), mild leukocytosis of 10.6 k/mm^3^ (range 4–10.5), and negative urinalysis. A peripherally inserted central catheter (PICC) was placed due to increasing vasopressor requirement, including now both norepinephrine and vasopressin.

During the patient's hospital admission, troponin levels have progressively increased, with sequential values of 32 → 75 → 737 ng/L. A transthoracic echocardiogram (TTE) was therefore performed and revealed a moderately reduced ejection fraction of 35%–40% and mid-to-distal anterior wall hypokinesis.

**Figure 3:**
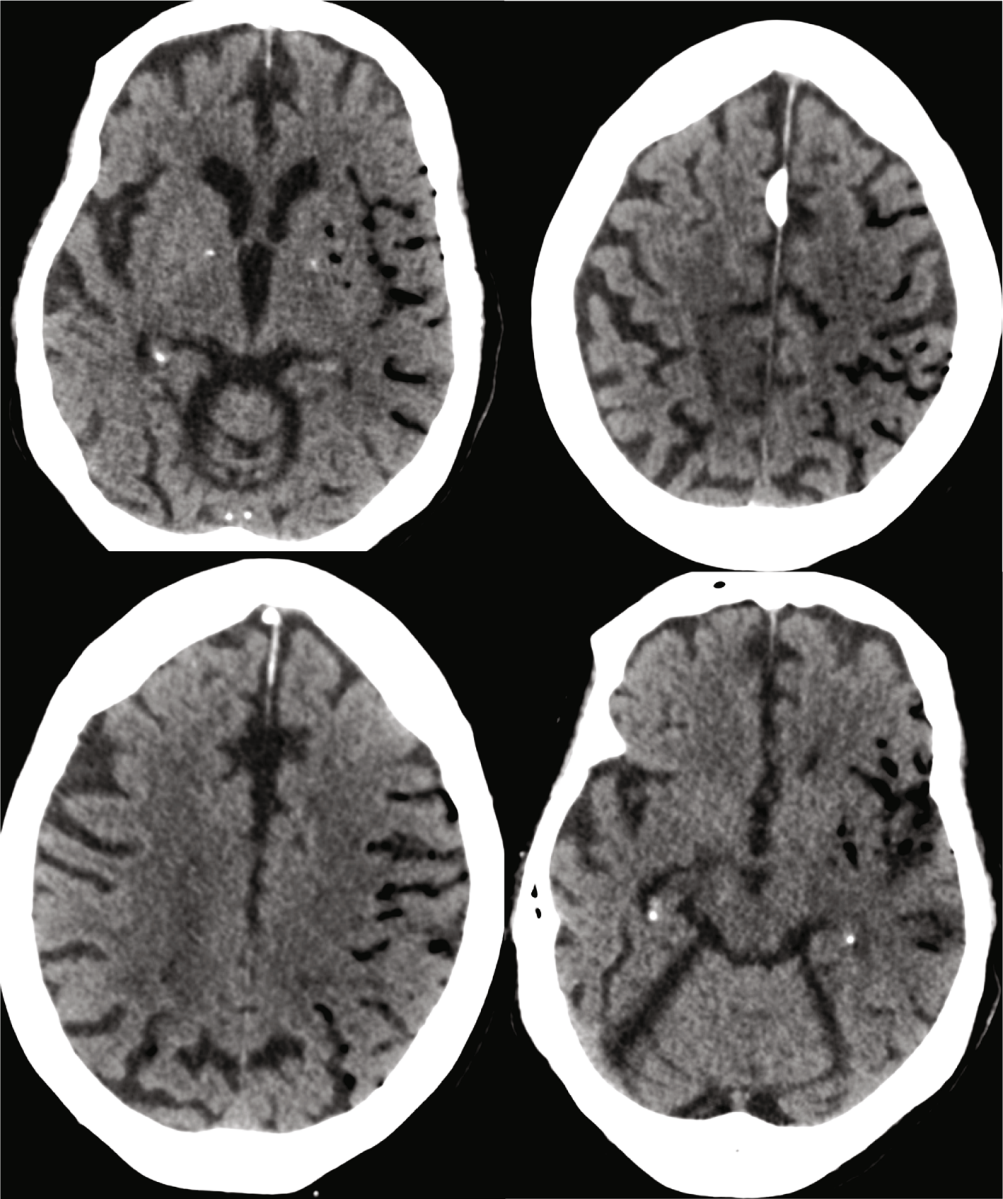
Multiple axial CT images of the head without contrast obtained after the patient's abrupt mental status changes demonstrate an extensive amount of intravascular air within the left MCA branches.

**Figure 4:**
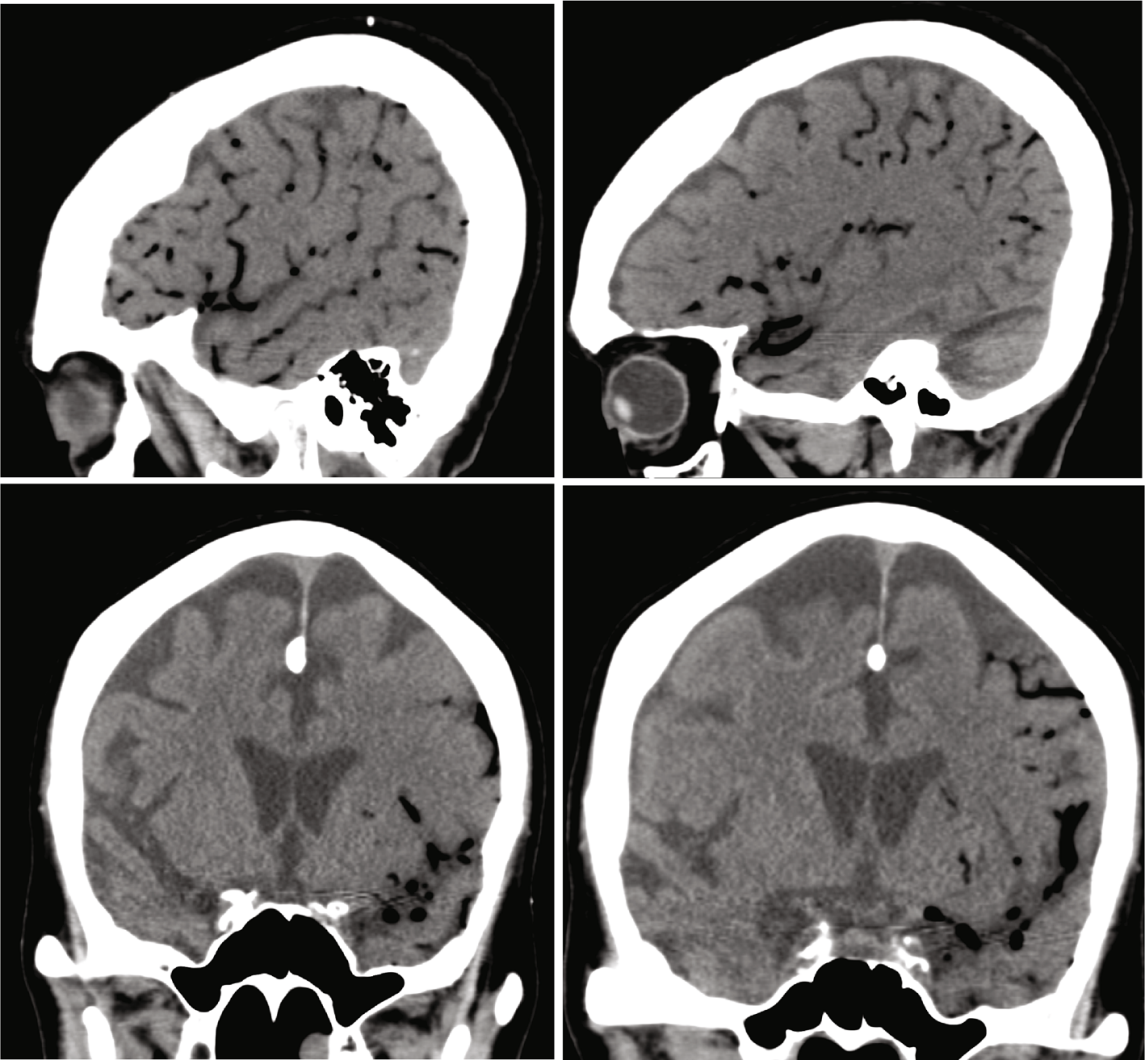
Sagittal and coronal CT images of the head without contrast demonstrate intravascular air within the left MCA distribution.

**Figure 5:**
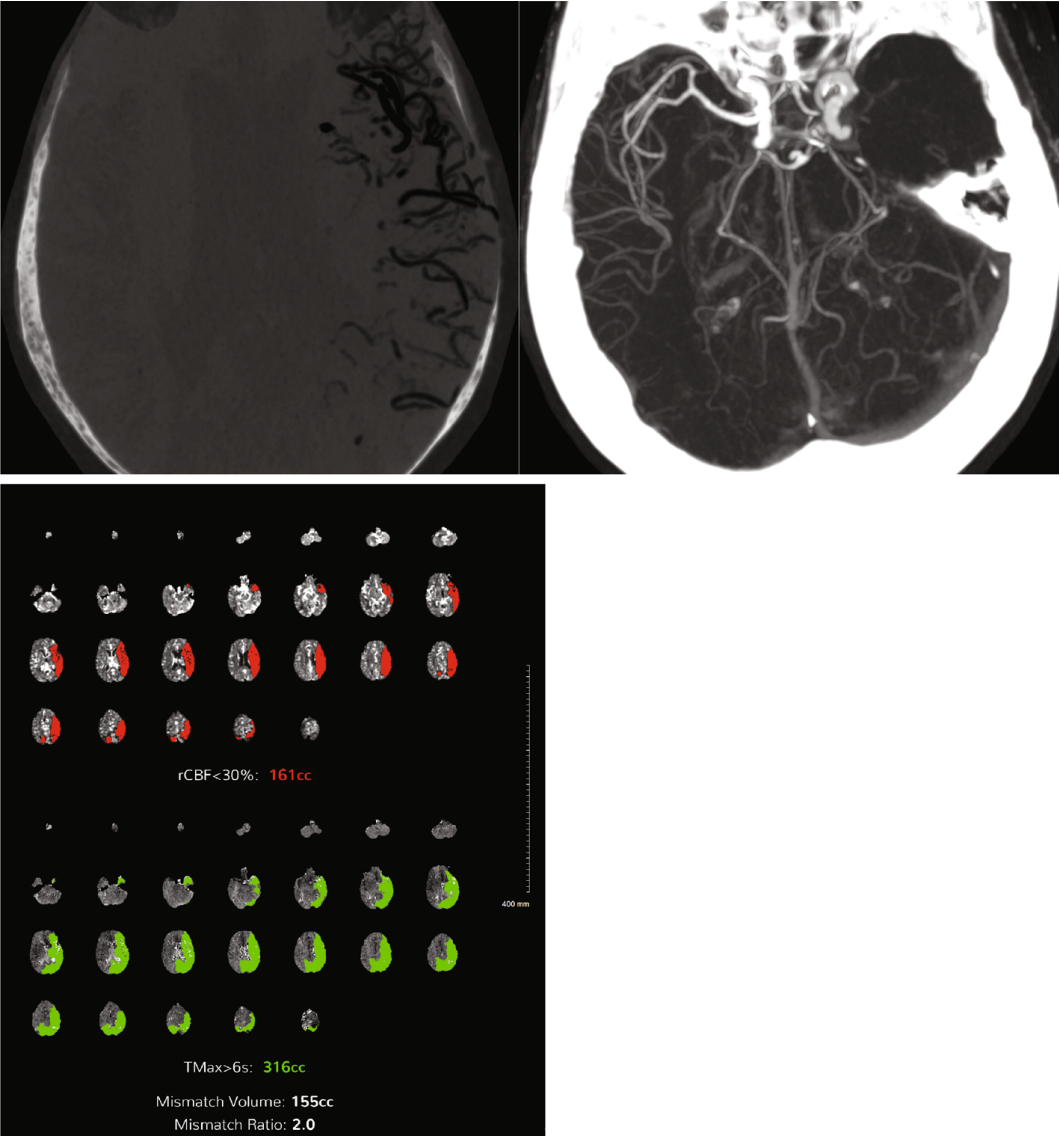
Maximal intensity image (MIP) and minimal intensity image (MIN) demonstrate complete occlusion of the left MCA vessels. CT brain perfusion software analysis demonstrates a large area of mismatch volume of 155 cc in the left MCA distribution.

On hospital Day 7, the patient was found unresponsive to verbal commands by the nursing staff, prompting activation of the medical emergency team. On examination, she demonstrated minimal facial grimace in response to sternal rub, hypotension (81/57 mmHg), and oxygen saturation > 90%. There were no known aspiration events or other acute precipitating factors for her sudden altered mental status. A repeat noncontrast CT of the head revealed extensive intravascular gas bubbles involving the majority of the left middle cerebral artery (MCA) vascular territory, consistent with a cerebral air embolism (Figures 3 and 4). Further imaging, including computed tomography angiography (CTA) of the head and neck and CT brain perfusion, confirmed a left MCA air embolism with a large acute left MCA territory ischemic infarct (Figure 5).

**Figure 6:**
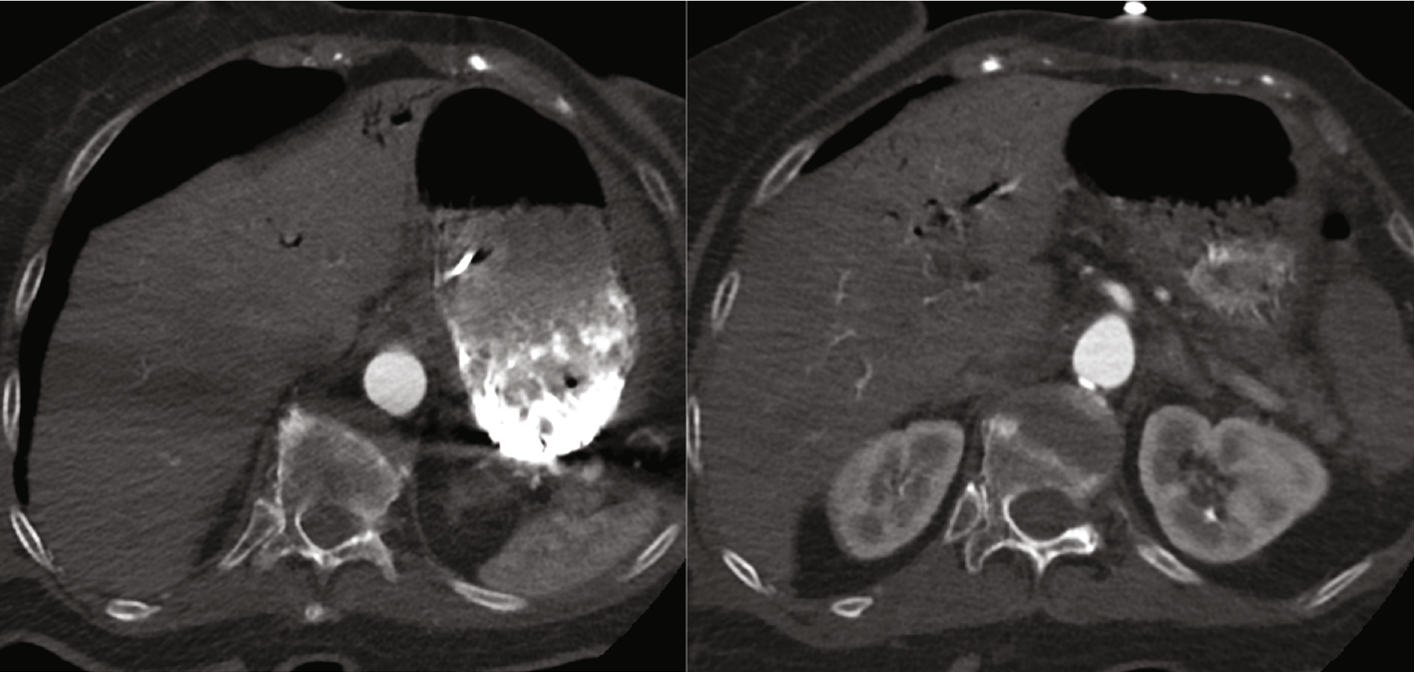
Axial CT images of the upper abdomen demonstrate portal venous gas in the liver hilum and left hepatic lobe.

The patient was intubated due to a Glasgow Coma Scale score of 4 and altered mental status. A repeat TTE with bubble study demonstrated bubbles in the right atrium, right ventricle, left atrium, and left ventricle, suggesting the presence of a pulmonary AVM or a PFO. Electroencephalogram was unremarkable, while CTA of the chest revealed portal venous gas in the left hepatic lobe, which was due to air emboli refluxing into the portal circulation, developing mesenteric ischemia in the setting of systemic hypoperfusion, or secondary to worsening sepsis-related complication (Figure 6). A follow-up CT of the head on the next day showed improvement in the intravascular gas within the left MCA territory without hemorrhagic conversion. The patient was placed in the left lateral decubitus, head-down position overnight.

## 3. Conclusions

To further investigate the cerebral air embolism, the PICC line was confirmed to be properly inserted into the venous circulation. Imaging findings and the case were reviewed by a multidisciplinary team, and the family was informed. Despite aggressive and prompt medical interventions, the patient's condition continued to decline, and she became unresponsive with no brainstem reflexes after sedation was discontinued. Considering her prognosis, care goals were transitioned to comfort measures.

## 4. Discussion

Cerebral air embolism is a rare but potentially fatal condition, often arising from clinical iatrogenic causes [7]. Unlike solid emboli such as thrombus, plaque, or septic emboli, air emboli can travel retrograde against blood flow due to the lower specific gravity of gas, increasing the complexity of clinical presentation and management [6]. For air to reach the cerebral arterial system, a preexisting right-to-left shunt is required to bypass the pulmonary capillary filtration system [8–11]. In our case, the bubble study strongly suggested a right-to-left shunt, allowing the emboli to bypass the pulmonary capillary filter. Air in the cerebral arterial vascular system causes ischemia through vessel occlusion or an inflammatory cascade that causes thrombus formation [11]. The presence of air in the left MCA led to rapid neurological deterioration. Notably, the embolism was unilateral, which may reflect preferential flow dynamics or anatomical variations in cerebral vasculature. The left common carotid artery originates directly from the aortic arch, whereas the right arises from the brachiocephalic trunk; differences in vessel diameter, angulation, and flow patterns may direct emboli preferentially to one hemisphere. Another possible explanation for rising troponins is coronary air embolism causing myocardial infarction or secondary myocardial ischemia from systemic hypoperfusion.

In addition, our patient presented in hypovolemic shock, exacerbated by the presumed sepsis, requiring vasopressors. Elevated lactate levels and clinical signs of dehydration pointed toward hypovolemia. Hypovolemia in combination with the semiupright position during CVC manipulation has been well-documented as a predisposing factor for developing air embolism [12]. Retrospective studies identify neurological manifestations in nearly all cases of cerebral air embolism [8]. In a systematic review of 158 cases, there were 10 cases of iatrogenic air emboli following CVC insertion, 10 cases that were related to CVC manipulation, and four cases that were unrelated to CVC use [8]. Most cerebral air emboli were associated with CVC disconnection or leaks in the setting of a right-to-left vascular shunt. Large intracranial air volumes may result from incomplete occlusion or open infusion routes during line manipulation. Despite confirmed line placement, inadvertent air entry remains plausible in low venous pressure and positional contexts.

Commonly reported treatments include sealing the air entry site, aspirating air from the CVC, placing the patient in the Trendelenburg position, administering high-flow oxygen, and using hyperbaric oxygen therapy [8]. It is recommended to provide 100% oxygen to these patients, the left decubitus position, and the Trendelenburg position to prevent the air bubbles from entering systemic circulation [9]. The most effective treatments for venous air emboli are hyperbaric oxygen [9].

The pathophysiology of air embolism involves multiple potential pathways:

1. Pulmonary circulation: Air emboli can obstruct the right ventricular outflow tract or pulmonary capillaries, resulting in respiratory symptoms and hemodynamic instability [1, 3, 12].
2. Right-to-left shunt: A right-to-left shunt, most commonly from PFO or pulmonary AVM, can allow air to bypass the pulmonary filtration system and enter the systemic circulation. This can result in coronary, cerebral, renal, or distal extremity emboli, causing acute heart failure, altered mental status, cerebral edema, acute kidney injury, or extremity gangrene [5, 6, 12].
3. Retrograde venous ascent: Air can ascend retrograde into the cerebral venous system, especially in patients in a semiupright or sitting position, leading to neurological symptoms [2, 12].

In our patient, the combination of a suspected right-to-left shunt, semiupright position during CVC manipulation, and hypovolemic shock created conditions conducive to air emboli. Chest CTA did not definitively identify a pulmonary AVM; imaging was not performed using a dedicated AVM protocol, so pulmonary AVM could not be ruled out. The presence of portal venous gas further complicated the clinical picture, suggesting systemic hypoperfusion, sepsis, or retrograde air migration [13]. Portal venous gas may represent systemic venous air entry via the PICC line with retrograde venous air movement, or alternatively, mesenteric ischemia from systemic hypoperfusion due to shock and sepsis. We acknowledge the uncertainty in definitively linking portal venous gas to the PICC line.

Pulmonary AVMs are rare congenital or acquired anomalies of the pulmonary vasculature that create a direct right-to-left shunt from the pulmonary artery to the pulmonary vein, bypassing the capillary bed [14]. Clinical manifestations of pulmonary AVMs vary, depending on the degree of shunting, and may include cyanosis, clubbing, anemia, and hypoxemia [5]. The entry of gas into the venous system is typically due to trauma, iatrogenic complications, laparoscopic procedures, neurosurgical procedures, or decompression sickness [15]. In this case, pulmonary AVMs likely facilitated the retrograde movement of air into the systemic circulation, compounding the patient's clinical deterioration [10]. In addition, gas bubbles obstructing blood flow in the capillaries in the form of microbubbles may coalesce into a larger bubble causing distal obstruction of a larger vessel [15]. This process, when it occurs in the pulmonary capillaries, will subsequentially increase alveolar dead space and intrapulmonary right-to-left shunting. The end result of the presence of microbubbles in the venous circulation is either that they will partially dissolve in the blood, be filtered by the lung capillaries, or travel into the arterial circulation through a pulmonary AVM or a PFO [15]. When there is a right-to-left shunt, the venous air embolism can travel into the arterial circulation and cause end organ ischemia [15].

The suggested path of the air could explain the presence of portal venous gas. There are limited literature resources on if the possibility of portal vein gas causing pulmonary or systemic gas emboli, without the combination of having hepatic AVM in the setting of right-to-left shunt [15].

The most plausible explanation in this case is air entry via the PICC line, with systemic dissemination due to a right-to-left shunt (likely a PFO or pulmonary AVM). Portal venous gas might reflect mesenteric ischemia due to systemic hypoperfusion and sepsis or retrograde migration of air within the venous system. Further investigation (e.g., confirmation of PFO with transesophageal echocardiography) would be needed to definitively identify the shunting mechanism.

## Data Availability

Data sharing is not applicable to this article as no datasets were generated or analyzed during the current study.

## Nomenclature

CVC: central venous catheter
PVM: pulmonary arteriovenous malformations
PFO: patent foramen ovale
